# Acoustic Features for Identifying Suicide Risk in Crisis Hotline Callers: Machine Learning Approach

**DOI:** 10.2196/67772

**Published:** 2025-04-14

**Authors:** Zhengyuan Su, Huadong Jiang, Ying Yang, Xiangqing Hou, Yanli Su, Li Yang

**Affiliations:** 1 Laboratory of Suicidal Behavior Research Tianjin University Tianjin China; 2 Institute of Applied Psychology Tianjin University Tianjin China; 3 School of Education Tianjin University Tianjin China; 4 Xi'an Mental Health Centre Xi'an China

**Keywords:** suicide, crisis hotline, acoustic feature, machine learning, acoustics, suicide risk, artificial intelligence, feasibility, prediction models, hotline callers, voice

## Abstract

**Background:**

Crisis hotlines serve as a crucial avenue for the early identification of suicide risk, which is of paramount importance for suicide prevention and intervention. However, assessing the risk of callers in the crisis hotline context is constrained by factors such as lack of nonverbal communication cues, anonymity, time limits, and single-occasion intervention. Therefore, it is necessary to develop approaches, including acoustic features, for identifying the suicide risk among hotline callers early and quickly. Given the complicated features of sound, adopting artificial intelligence models to analyze callers’ acoustic features is promising.

**Objective:**

In this study, we investigated the feasibility of using acoustic features to predict suicide risk in crisis hotline callers. We also adopted a machine learning approach to analyze the complex acoustic features of hotline callers, with the aim of developing suicide risk prediction models.

**Methods:**

We collected 525 suicide-related calls from the records of a psychological assistance hotline in a province in northwest China. Callers were categorized as low or high risk based on suicidal ideation, suicidal plans, and history of suicide attempts, with risk assessments verified by a team of 18 clinical psychology raters. A total of 164 clearly categorized risk recordings were analyzed, including 102 low-risk and 62 high-risk calls. We extracted 273 audio segments, each exceeding 2 seconds in duration, which were labeled by raters as containing suicide-related expressions for subsequent model training and evaluation. Basic acoustic features (eg, Mel Frequency Cepstral Coefficients, formant frequencies, jitter, shimmer) and high-level statistical function (HSF) features (using OpenSMILE [Open-Source Speech and Music Interpretation by Large-Space Extraction] with the ComParE 2016 configuration) were extracted. Four supervised machine learning algorithms (logistic regression, support vector machine, random forest, and extreme gradient boosting) were trained and evaluated using grouped 5-fold cross-validation and a test set, with performance metrics, including accuracy, *F*_1_-score, recall, and false negative rate.

**Results:**

The development of machine learning models utilizing HSF acoustic features has been demonstrated to enhance recognition performance compared to models based solely on basic acoustic features. The random forest classifier, developed with HSFs, achieved the best performance in detecting the suicide risk among the models evaluated (accuracy=0.75, *F*_1_-score=0.70, recall=0.76, false negative rate=0.24).

**Conclusions:**

The results of our study demonstrate the potential of developing artificial intelligence–based early warning systems using acoustic features for identifying the suicide risk among crisis hotline callers. Our work also has implications for employing acoustic features to identify suicide risk in salient voice contexts.

## Introduction

### Background

Suicide is a global public health issue. According to the World Health Organization in 2019, approximately 703,000 people died by suicide each year worldwide [1]. Therefore, massive studies have been conducted to identify suicidal risks, which is crucial for preventing and reducing suicide [2-4]. However, most previous works relied on the language content, including clinical interview and assessment using self-report scales, when identifying suicidal risks [5-7]. In this study, we aimed to investigate how to use acoustic features to identify suicide risk in the context of crisis hotlines. In fact, speech has been used for suicide diagnosis since decades [8]. For example, studies utilizing linguistic analysis have shown that when a person is suicidal, their speeches become hollow and toneless [9]. However, such a manual speech analysis approach cannot be applied in large-scale research and clinical environments [10]. Now these acoustic changes can be well captured using acoustic speech features [11]. Moreover, with the development of artificial intelligence technologies such as machine learning, it is possible to analyze highly complex patterns in acoustic features [12]. Using artificial intelligence to automatically analyze features can help us move from a clinical practice model that relies solely on clinician judgment to an evidence-based medicine model based on data measurements [13].

Some studies have used acoustic features as indicators to automatically identify suicidal ideation and to determine suicide risks in populations such as veterans, active duty soldiers, and university students. The audio materials in these studies are mostly derived from laboratory interviews or spontaneous recordings such as audio diaries [14-23]. In the context of collecting such audio materials, acoustic information is less important due to the assistance of nonverbal information and suicide screening scales. However, voice messages become particularly important in special contexts such as in crisis hotline calls [24].

As a suicide prevention method, crisis hotlines play a crucial role in early detection and response to suicide risk [25]. The World Health Organization estimates that there are more than 1000 crisis hotlines worldwide. Crisis lines provide a confidential and stigma-free alternative for individuals who are suicidal and may not seek help from traditional health services, family, or friends, or who have not disclosed their suicidal thoughts to professionals, thereby reaching those who are otherwise unreached for their mental health struggles [26]. However, accurately assessing suicide risk in a crisis hotline is a difficult task. Due to their anonymous, time-limited, and typically single-occasion nature, crisis hotline counselors cannot predict or control the type of calls they receive and are expected to respond as quickly as possible to the risks of callers [26]. Furthermore, individuals identified as high-risk callers in crisis lines are significantly more likely to engage in subsequent suicidal behavior than those identified as low risk. Recognizing the suicide risk of callers is the first critical step to manage the risk for crisis hotline counselors. When the crisis hotline counselor realizes that the caller is at high risk, more urgent intervention strategies are employed to help the caller manage the risk [27]. Simply identifying the presence of suicidal ideation in callers is not sufficient—a more thorough assessment by crisis hotline counselors is required [28]. Unlike risk assessments in the form of face-to-face interviews, crisis hotline counselors are unable to observe nonverbal communication cues [29]. Therefore, crisis hotline counselors rely solely on vocal communication, and they have to be highly attuned to every sound, silence, inflection, and quality of speech, including tone, pitch, and speed [30]. This will undoubtedly add to the burden of the counselors. It would be helpful if acoustic information could be used to assist the counselor in risk assessment and then management. Therefore, approaches or techniques that can identify suicide risk automatically based on the acoustic characteristics of the caller is promising.

Although there have been studies [9,14-16,19,20,22,24] exploring the effectiveness of acoustic features in suicide risk identification, they are understudied in crisis hotlines. Recorded calls to crisis hotlines are characterized by low sampling rates (8 kHz) and poor recording environments. These limitations pose substantial challenges for the acoustic analysis of the data [31,32]. The study by Iyer et al [33] is one of the few studies that have tested the feasibility of acoustic features for suicide risk identification in hotline callers. Their findings suggested that acoustic features have the potential to be considered as biomarkers of suicide risk in callers [33]. However, because they used the uncommon method of independent analysis of speech frames and did not validate the model on an independent test set, the results of their study require further validation.

### Objective

This is a retrospective machine learning study. The purpose of this study was to first train a machine learning model by using speech material from a crisis hotline and to test the performance of the model on an independent test set. In doing so, we aim to test which machine learning model is more suitable to be applied for the risk identification of hotline callers. Second, considering the characteristics of the sampling rate and the recording environment of the hotline speech material, this study further investigates whether advanced acoustic features (high-level statistical functions [HSFs]), which have better recognition performance than basic acoustic features, contribute to the recognition performance of the machine learning model [34-36].

## Methods

### Study Materials and Clinical Assessment

A total of 525 calls were selected from the records of a psychological assistance hotline in a province in northwest China between January 2022 and March 2023. These calls were identified as involving suicide-related conversations between the counselor and the caller. The callers included both adolescents and adults (aged 12 years and older). The counselor assessed the caller’s suicide risk according to the “suicidal thoughts and plans” entry in the risk assessment criteria for Chinese crisis hotlines [28]. Specifically, callers exhibiting suicidal ideation without a suicide plan were categorized as low risk; callers presenting with suicidal ideation accompanied by a suicide plan, and who were in the process of executing or preparing to engage in suicidal behavior within the subsequent 72-hour period, or had a recent history of a suicide attempt within the preceding 2 weeks were designated as high risk.

To verify the accuracy of the initial classifications by the hotline counselor, we recruited another sample of raters to rate the callers’ suicide risk in each recording. A total of 18 risk raters with a background in clinical psychology and experience working with crisis hotlines were recruited. They were asked to rate the recordings included in the study, according to the “suicidal thoughts and plans” entry in the risk assessment criteria for Chinese crisis hotlines. Each rater rated 10 randomly selected suicide risk recordings to assess interrater agreement before engaging in assessment. The interrater reliability (κ) of the clinical assessment conducted by a team of 18 raters was 0.771 for a random selection of 10 recordings. This value represents that there is a high degree of rater agreement among the raters [37].

The assessment of each recorded suicide risk call was conducted by 2 independent raters. Callers whose statements were deemed indicative of suicide risk by both raters and who exhibited a similar level of suicide risk, as indicated by their respective assessment ratings, were included in this study. Risk assessors were asked to make notes on the point in time of the dialogue where suicide-related themes (including suicidal ideation, suicide planning, history of suicide attempts, and ongoing suicidal behaviors) occurred in the recording.

### Data Exclusion

Risk recordings were excluded if they did not adhere to the established assessment process. This included instances where the caller’s suicide plan, preparation, or other relevant factors were not adequately assessed after the disclosure of suicidal ideation. Additionally, recordings where the caller’s expression was unclear or insufficient for risk assessment were excluded. The final screening resulted in 164 recordings where the caller’s expression was sufficiently clear to indicate their level of risk. Of these, 102 calls were assessed as low risk and 62 as high risk. The authors extracted segments of suicide-related expressions at specific time points where raters identified the occurrence of suicide-related conversations. All audio clips of suicide-related expressions with a duration of 2 seconds or more were manually intercepted [38,39]. We obtained a total of 273 clips (132 clips for high risk and 141 clips for low risk). The high risk and low risk segments were used in subsequent model training and evaluation [33].

### Preprocessing of Call Recordings

All call recordings were originally saved in MP3 (MPEG-1 audio layer 3) format with a sampling rate of 8 kHz, a bit depth of 32 bits, and in dual channel. The suicide-related clips labelled by the risk assessors were checked and relistened to by the first author, and complete sentences of suicide-related expressions were intercepted as audio-recorded material. We also removed the crisis hotline counselor’s voice channel and only kept the caller’s voice. After removing the nonspeech fragments from the first and end points of the clip by using voice activity detection, we converted the audio files to WAV (waveform audio file) format.

### Feature Extraction

#### Basic Acoustic Feature Extraction

Spectrum features, quality features, and rhythm features were extracted for each segmented utterance and averaged over the entire time interval [24,40]. The spectral characteristics of the audio signal were captured through a 39-dimensional Mel Frequency Cepstral Coefficients (MFCCs) representation [41,42]. The quality attributes of the signal are represented by the center frequency and bandwidth of the first 3 formants, in addition to jitter and shimmer measurements [20,43]. Rhythmic aspects of the speech are quantified through metrics such as the duration of effective speech segments, fundamental frequency (pitch), short-time energy, and sound pressure level [16].

#### Advanced Feature Extraction

##### Open-Source Speech and Music Interpretation by Large-Space Extraction

OpenSMILE (Open-Source Speech and Music Interpretation by Large-Space Extraction), an open-source tool and a robust platform for the extraction of acoustic features, can take the original waveform signal of a sound signal in a time series as input and output the names and values of the corresponding acoustic features [44]. We employed the ComParE 2016 configuration profile to extract the 6373-dimensional features of this feature set. The ComParE 2016 feature set incorporates more combinations of low-level descriptors and functionals than the basic acoustic feature set [45]. This large feature set provides a quantification of voice characteristics that is more comprehensive than ever before, and it has demonstrated effectiveness in the fields of emotion and personality trait recognition [39,46].

##### Mutual Information

To avoid overfitting the training model and to compare it with models constructed from basic acoustic features, this study uses the mutual information method to reduce the number of feature dimensions of the advanced feature set to be consistent with the basic acoustic feature set. The mutual information method computes the degree of mutual information, which indicates the dependency between features and discrete binary labels. The higher the mutual information degree, the stronger the dependency between the feature and the label, and therefore, the more useful it is for model recognition [47]. Mutual information has been employed for dimensionality reduction within high-dimensional feature spaces relevant to suicide acoustic studies [36]. Extraction was performed using Python (version 3.6), and the following packages were used to extract the acoustic features: *librosa* (version 0.7.2), *NumPy* (version 1.19.5), and *pandas* (version 1.1.5). Normalization or filtering was not performed during the preprocessing stage.

### Machine Learning Methods

In mental health research, traditional machine learning methods are frequently favored due to their interpretability and suitability for smaller datasets [48]. For the problem of risk identification in the field of suicide, supervised machine learning methods are usually the most applicable [12]. In consideration of the scale of the dataset employed in this study and the research questions posed, we utilized supervised machine learning as the modality of data analysis. We employed 4 supervised machine learning algorithms: logistic regression, support vector machine, random forest, and extreme gradient boosting [36].

**Figure 1:**
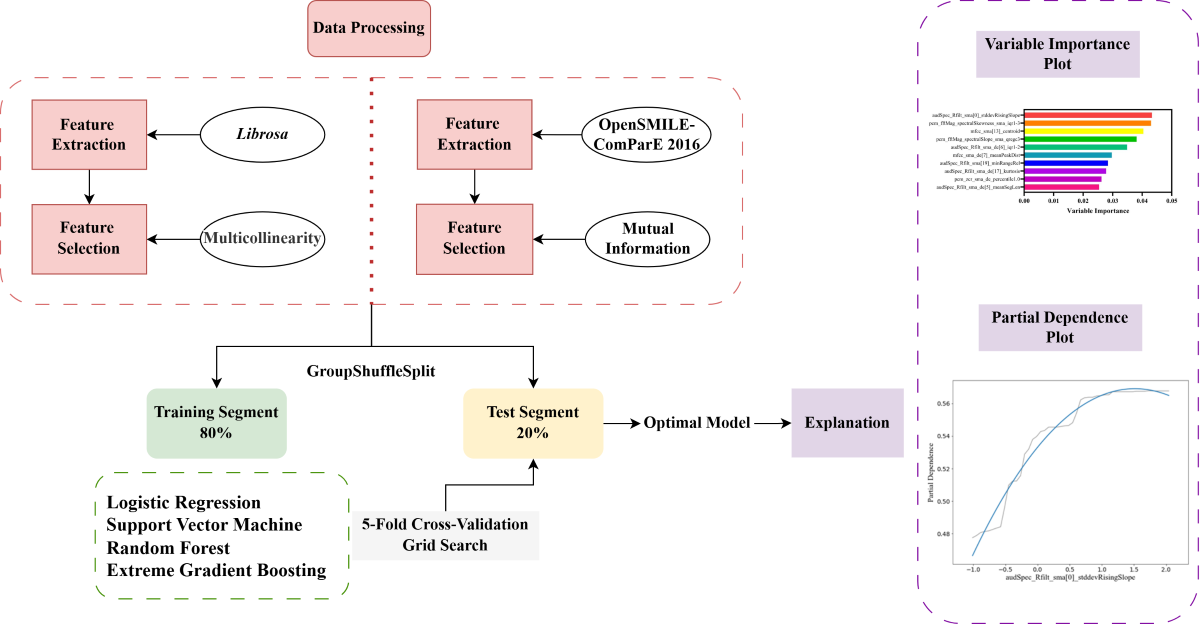
Flowchart of the machine learning analysis process for suicide risk assessment in crisis hotline callers, including data preprocessing, model training, and evaluation. OpenSMILE: Open-Source Speech and Music Interpretation by Large-Space Extraction.

The entire machine learning analysis process is shown in Figure 1. The data were divided into training and test sets using the GroupShuffleSplit method, with an 80:20 ratio. This prevents the information leakage of the same incoming call by dividing multiple segments of the same call into training and test sets at the same time. In the training set, we employed a grouped 5-fold cross-validation approach with a grid search strategy to optimize the model parameters [49]. The optimal combination of parameters was selected based on the training set, and its performance was subsequently evaluated using the test set.

We used accuracy to evaluate the overall recognition performance of the model and *F*_1_-score, recall, and false negative rate (FNR) to evaluate the model’s detection performance for high-risk callers. Higher values for accuracy, *F*_1_-score, and recall metrics imply a superior performance of the model in accurately identifying instances of suicide risk. Reduced values of FNR indicate a diminished likelihood of misclassifying high-risk individuals as low risk within the caller population. True positive (TP) is the number of samples that were correctly classified as belonging to the high-risk class. False positive (FP) refers to the number of samples that were incorrectly classified as high risk. False negative (FN) is the number of high-risk samples that were misclassified as low risk. True negative (TN) is the number of samples that were correctly classified as low risk [50]. The evaluation metrics are defined as follows:

Accuracy = (TP + TN) / (TP + FP + TN + FN)

*F*_1_-score = 2TP / (2TP + FP + FN)

Recall = TP / (TP + FN)

FNR = FN / (TP + FN)

### Ethics Approval

This study has been approved by the institutional review board of Tianjin University (2024-453). The researchers confirm that all stages of this study were conducted in accordance with the ethical standards set forth by the Helsinki Declaration, as revised in 1989. Prior to being connected with a hotline operator, callers were informed via an automated message that their calls would be recorded and that any data obtained from these calls would be treated in accordance with the tenets of confidentiality and analyzed in an anonymized manner. All data have been anonymized, and any private information related to the caller has been removed.

## Results

### Descriptive Statistics for Suicide-Related Expressions

The gender and age of the callers of the 273 suicide-related statements are shown in Table 1. We first conducted chi-square tests to examine whether the gender and age range ratios differed in high and low suicide risk conditions.

**Table 1:** Demographic characteristics (sex and age) of the callers with suicide-related expression segments included in this study (N=273).

Characteristic		High risk of suicide, n (%)	Low risk of suicide, n (%)
Number of segments		132 (48.4)	141 (51.6)
**Sex**			
	Male	62 (47)	48 (34)
	Female	70 (53)	93 (66)
**Age (years)**			
	12-18	57 (43.2)	36 (25.5)
	19-34	58 (43.9)	74 (52.5)
	35+	6 (4.5)	20 (14.2)
	Unspecified	11 (8.3)	11 (7.8)

A significant association was observed between gender and risk category (*χ^2^*_1_=6.1; *P*=.01). The low-risk group showed higher female representation, while the high-risk group had a balanced gender ratio. With regard to age, the age distribution differed significantly between risk groups (*χ^2^*_3_=18.2; *P*<.001). The proportion of callers aged 12-18 years within the high suicide risk caller segment was higher than that within the low suicide risk segment (*χ^2^*_1_*=*9.5; *P*=.002). Additionally, the proportion of callers older than 35 years within the high suicide risk caller segment was lower than that within the low suicide risk segment (*χ^2^*_1_=7.4; *P*=.007). There were no significant differences in other age ranges. An independent sample 2-tailed *t* test was conducted to compare the duration of suicide-related utterances in high-risk and low-risk calls. There was no significant difference in the duration for high-risk (mean 6.470, SD 5.365) and low-risk (mean 6.262, SD 4.378) conditions (*t*_271_=–0.351; *P*=.73).

### Suicide Risk Recognition With Basic Acoustic Features

#### Feature Selection

We extracted spectrum features, quality features, and rhythm features totaling 53 dimensions and performed multicollinearity diagnosis on these features. Fifty-dimensional basic acoustic features were included in this study after excluding 3 dimensions with a variance inflation factor greater than 10 (sound pressure level, MFCC1, and MFCC2). Multimedia Appendix 1 and Multimedia Appendix 2 present the feature information that survives multicollinearity diagnostics.

#### Machine Learning Models for Basic Acoustic Features

**Figure 2:**
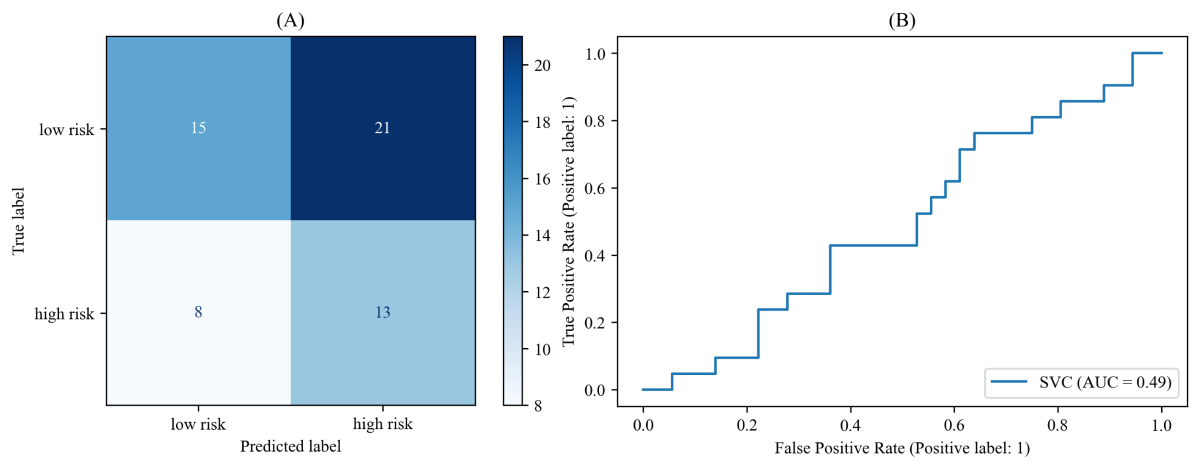
(A) Confusion matrix and (B) receiver operating characteristic curves for a basic acoustic feature caller risk classification model based on support vector machines. AUC: area under the curve; ROC: receiver operating characteristic; SVC: support vector classification.

The results of the recognition models trained with basic acoustic features are presented in Figure 2 and Table 2. Support vector machine demonstrated the most optimal performance in the model that was trained using the basic acoustic feature set, with an accuracy of 0.49. The model achieved an *F*_1_-score of 0.47, recall of 0.62, and FNR of 0.38. The model demonstrated a 49% accuracy rate in identifying high/low risk, which is not significantly superior to the accuracy expected by chance. Therefore, machine learning models incorporating advanced acoustic features are required, which will be described in the next section.

**Table 2:** Performance comparison of the 4 machine learning models using basic acoustic features for suicide risk classification in crisis hotline callers.

Machine learning model (testing sets)	Accuracy	*F*_1_-score	Recall	False negative rate
Logistic regression	0.44	0.36	0.43	0.57
Random forest	0.44	0.37	0.48	0.52
Support vector machine	0.49	0.47	0.62	0.38
Extreme gradient boosting	0.38	0.31	0.38	0.62

### Suicide Risk Recognition With Advanced Acoustic Features

#### Feature Selection

In this study, 50 advanced acoustic features with the highest mutual information were selected in alignment with the number of basic acoustic features from a set of 6373-dimensional features. A multicollinearity test was performed on the 50-dimensional features after dimensionality reduction, and it was found that none of them had multicollinearity. The details of the 50 advanced acoustic features are presented in Multimedia Appendix 3.

#### Machine Learning Models for Advanced Acoustic Features

**Figure 3:**
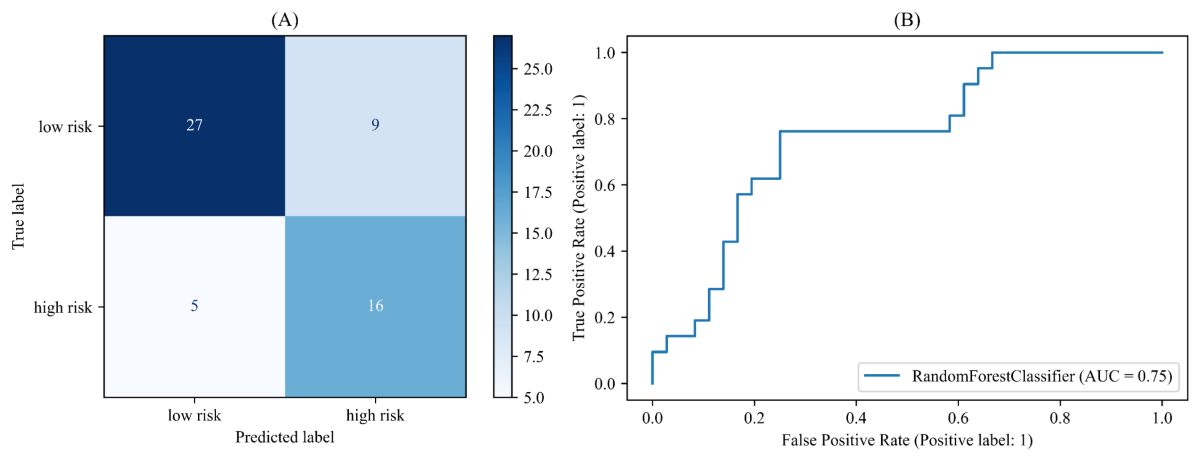
(A) Confusion matrix and (B) receiver operating characteristic curves for random forest–based high-level statistical function acoustic features caller risk classification model: area under the receiver operating characteristic curve. AUC: area under the curve; ROC: receiver operating characteristic.

The results of the recognition models trained with advanced acoustic features are presented in Figure 3 and Table 3. Random forest demonstrated the most optimal performance in the model that was trained using the advanced acoustic feature set, with an accuracy of 0.75. The model achieved an *F*_1_-score of 0.70, recall of 0.76, and FNR of 0.24. The model trained with advanced acoustic features showed higher recognition performance than that trained with basic acoustic features.

**Table 3:** Performance evaluation of the 4 machine learning models utilizing advanced acoustic features for suicide risk prediction in crisis hotline callers.

Machine learning model (testing sets)	Accuracy	*F*_1_-score	Recall	False negative rate
Logistic regression	0.61	0.24	0.48	0.52
Random forest	0.75	0.70	0.76	0.24
Support vector machine	0.58	0.18	0.43	0.57
Extreme gradient boosting	0.63	0.55	0.62	0.38

**Figure 4:**
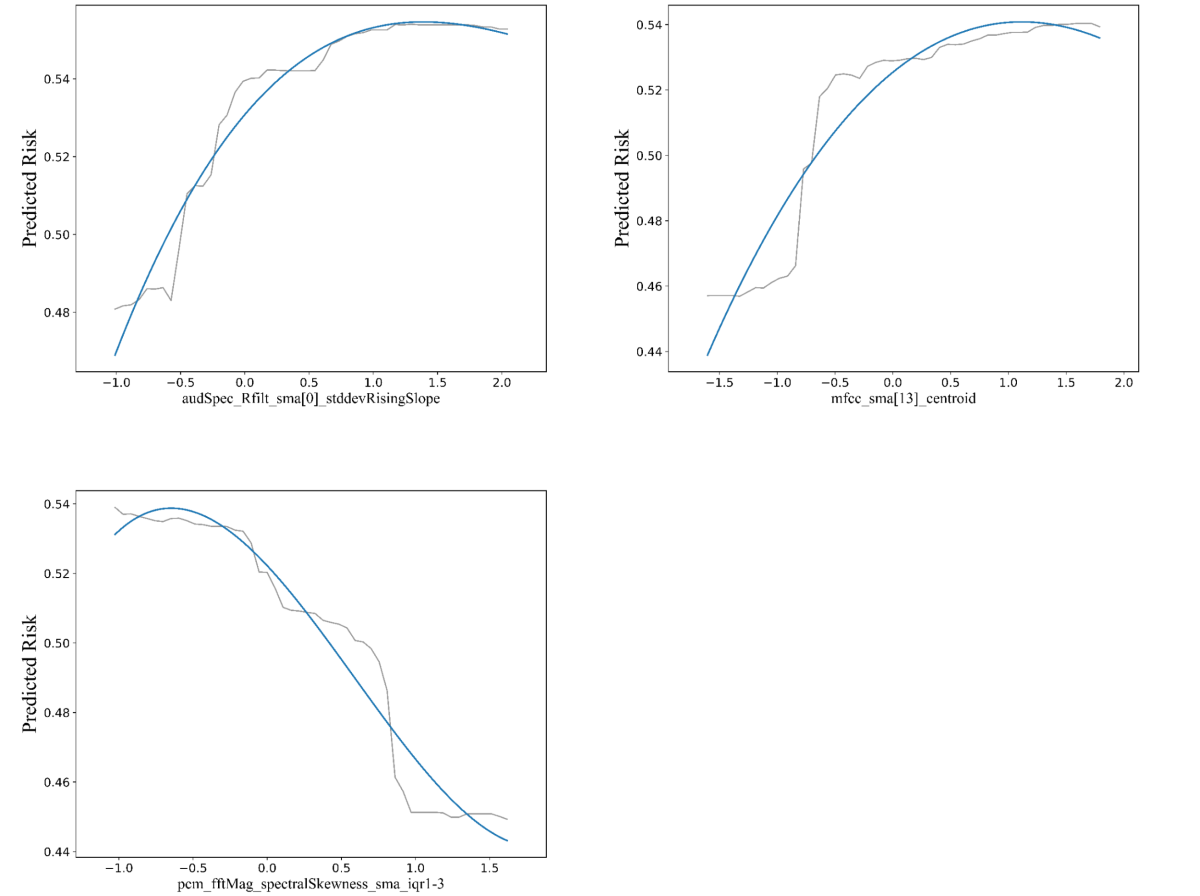
Partial dependence plots of the top 3 most important variables in the random forest model for suicide risk assessment using high-level statistical function features.

Considering that random forest achieved the best suicide risk identification performance by using the downgraded HSF feature, we chose to explore the relationship between the acoustic features and the degree of suicide risk in it by using variable importance plot versus partial dependence plot (PDP). The importance of the features in the classification model is illustrated in Multimedia Appendix 4. The top 3 most important features of the classification model were audSpec_Rfilt_sma [0]_stddevRisingSlope (SRRS), pcm_fftMag_spectralSkewness_sma_iqr1-3 (PMSS), and mfcc_sma [13]_centroid (MFSC). SRRS is the standard deviation of the rising slope of the first element in the simple moving average (SMA) of the audio spectrum after being filtered. PMSS is the spectral skewness, calculated using SMA of the magnitude of the Fast Fourier Transform of the Pulse Code Modulation signal, with IQR spanning from the first to the third quartiles. MFSC is the centroid of the 13th coefficient in the MFCC feature set, smoothed with SMA. The PDPs of the most important variables in the classification model illustrate the relationship between the probability of being classified as high-suicide risk (y-axis) and the acoustic features (x-axis). Figure 4 illustrates the nonlinear relationship between the 3 most significant acoustic features in the random forest model and the probability of being classified as high risk, along with the corresponding feature. The PDPs for the 2 variables SRRS and MFSC exhibit a similar trend, whereby the probability of being categorized as high risk increases in tandem with the value of the variable. The highest probability of being categorized as high risk is observed when the value of the variable reaches a value between 1 and 1.5, followed by a slight decline. In contrast, the PMSS feature exhibits a divergent trend, with the probability of being classified as a high-risk caller demonstrating a slight increase and reaching a maximum as the variable value increases within the interval between –1 and –0.5. The probability of being categorized as a high-risk caller tends to decrease in intervals where the value of the variable is greater than –0.5 and less than 1.5.

## Discussion

### Main Findings

This study tests the feasibility of using acoustic features to identify the suicide risk of crisis hotline callers. In doing so, we collected suicide-related calls to a crisis hotline and analyzed the acoustic features of high-risk versus low-risk suicidal calls. We extracted different sets of acoustic features by using 2 methods. First, the Python-based *librosa* library was used as in existing studies [24] and the basic acoustic features were extracted and averaged over the whole time interval. The second method that we used was OpenSMILE, an audio feature extraction tool, to extract 6373-dimensional HSFs for hotline speech segments and to perform dimensionality reduction by using the mutual information method. We used 4 machine learning algorithms to train models on each of the 2 feature subsets and to compare performance between algorithmic models. In the subset of basic acoustic features, the 4 machine learning models performed poorly, with the best performing support vector machine achieving only 49% recognition accuracy. In the HSF feature subset, all 4 machine learning algorithms had better accuracy. The classification performance of the random forest model was much better than all the other 3 algorithms, reaching 75% accuracy, that is, random forest model using a subset of HSF features is likely to be a feasible approach to identify the suicide risk of hotline callers. We found that voice characteristics, especially the HSF features, have the potential to serve as an objective indicator for identifying callers’ suicide risk in a crisis helpline. We also agree with Draper et al [51] that constructing such a classification model for acoustic information is not designed to replace the counselor’s judgement, but it may assist the counselor in assessing short-term warning signs for suicide.

### Strengths

We obtained and analyzed authentic caller audio clips from a crisis hotline, which offers a high degree of ecological validity. Given the dearth of research on speech material in the context of crisis hotlines [33], this study makes a valuable contribution to the automated quantitative analysis of voices in this context. The majority of previous studies utilized automatic speech analysis of acoustic features for the detection of suicidal ideation [15,22,52-55]. However, in the context of crisis hotline services, the mere identification of the presence or absence of suicidal ideation expressions in callers is often insufficient. This study identifies and classifies low-risk and high-risk callers to the crisis hotline, going beyond relying solely on language in recognizing suicide risk.

Additionally, rigorous exclusion criteria were employed to exclude all callers with ambiguous suicide risk levels. Two trained raters independently reassessed each caller’s recordings to obtain more accurate clinical assessment labels. As the clinical assessment was based on suicide-related expressions, only speech segments of suicide-related expressions were included in this study. High quality data, with redundancy and irrelevant speech segments removed and accurately annotated, help improve the classifier’s recognition performance [56]. This allowed for a more detailed examination of how well machine learning models constructed solely on acoustic features match with accurate clinical assessments.

In this study, we employed an approach that can directly extract acoustic features from speech segments, differing from Iyer et al’s [33] frame-based analysis of speech. The method we used is conducive to the prevention of the loss of feature information that might otherwise result from the exclusion of silent frames. Additionally, building on the foundational research by Iyer et al [33], we conducted validation on a test set that is independent of the training set, corroborating that acoustic features can indeed serve as markers for identifying the risk of suicide in hotline callers.

The machine learning model we trained using the basic set of acoustic features extracted from previous research in laboratory interview scenarios did not show good performance on the test set. This may be due to the quality of the recording material. The sampling rate for call recordings is 8 kHz, whereas the sampling rate of microphone equipment for interview recordings is usually several times higher [24]. Therefore, we analyzed the advanced acoustic features of hotline callers. In alignment with the findings of previous studies and the hypotheses proposed, the more comprehensive advanced statistical function features demonstrated superior performance in the risk classification of crisis hotline callers. This may assist in circumventing the constraints imposed by the low sampling rate of hotline audio recordings [34,35,39]. Furthermore, the random forest model trained on the subset of HSF features demonstrated the highest recognition performance. This is also consistent with that reported in previous studies, where tree-based models have been found to perform better than other machine learning models in suicide voice-related databases [15].

We also conducted further model interpretation, highlighting the top 10 features that significantly influenced the model’s classification accuracy. PDPs were then used to present the relationship between the 3 most important dimensions and model categorization. The 3 most important variables in the random forest model trained with advanced acoustic features were SRRS, MFSC, and PMSS. Among them, SRRS and MFSC are 2 typical features of the RASTA (Reliable And Smooth Template Algorithm) style-filtered auditory spectra and MFCC, respectively, which are the most relevant acoustic features of the valence dimension [57], evaluating the pleasure level of the emotion [58]. In our study, this meant that although both low-risk and high-risk callers made suicide-related statements, there were some differences in their emotions. PMSS was associated with increased vocal effort, hyperfunction of the neck muscles, and potential laryngeal compression [59,60]. Such an increase in vocal effort also means that low-risk and high-risk callers have different stress levels [61].

Given the aforementioned strengths, our work has implications for developing a theory or framework to identify the suicide risk of crisis line callers. On the one hand, the advanced features highly related with suicide risk shed light on the developing framework to identify suicidal callers in crisis hotlines. As predicted, callers with suicidal risk could be recognized quickly according to their voice features. On the other hand, we found that the approach of the random forest model based on HSF features is the optimal. Follow-up work can use such models to analyze the HSF features to replicate and extend our work. What’s important, approaches may also be developed to automatically identify suicidal callers according to their voice, which will be helpful and valuable for timely prevention and intervention through crisis hotlines. Such an automatic procedure can also help compensate for the manual limitations of crisis hotlines.

### Limitations and Future Directions

Our work also has limitations. First, we did not control for characteristics that could potentially diminish the classification performance of the machine learning model [62,63]. Our study includes all low-risk and high-risk callers because the sample sizes for subgroups based on demographic features such as age and gender were insufficient for independent analysis. As the acoustic features vary across different age and gender groups, our findings may be limited by not controlling for such demographic variables, which is awaiting further explorations in future work. Second, we only utilized machine learning as the data analysis method, being constrained by the limited sample size of the study. Studies have applied deep learning methods to identify depressed patients, achieving high accuracy in model performance [64]. Future research can utilize deep learning to explore more complex relationships between acoustic features and suicide risk within larger datasets. Third, the content of the recordings was not considered in this study. The narrative content of crisis hotline communications is critical, as it is the primary reference for assessing the caller’s risk assessment. It has been demonstrated that the fusion of acoustic and textual features through multimodal techniques enhances the accuracy of recognition [15]. It is expected that, in the future, means of combining chat text with acoustic information will help to develop more refined models of risk assessment for hotline callers [65]. Fourth, in light of the relatively low base rate of suicide, the overall positive predictive value for the identification of high-risk callers is low [28]. This implies that crisis hotline counselors must remain vigilant to the potential for misclassification, that is, high-risk callers may be inaccurately assessed as low risk, while low-risk callers might be mistakenly evaluated as high risk. Consequently, the acoustic-based risk assessment should not be used in isolation but rather as a complementary tool to other risk assessment methods employed by counselors.

### Conclusion

This study suggests that voice characteristics are promising objective indicators for detecting suicide risk among crisis helpline callers. We demonstrated that HSF features can be employed to identify suicide risk in crisis helpline callers, especially based on the random forest model (a typical machine learning model). Although further external validation and methodological optimization are needed to validate and extend the findings of this study, our work holds promise for real-time assessment of high-risk callers by using acoustic features.

## Appendix

Multimedia Appendix 1 Fifty basic acoustic feature dimensions included in this study.

Multimedia Appendix 2 Ten-dimensional acoustic features with the highest variance inflation factor identified from the 50-dimensional basic acoustic feature set for suicide risk assessment. MFCC: Mel Frequency Cepstral Coefficient; VIF: variance inflation factor.

Multimedia Appendix 3 Fifty high-level statistical function features included in this study.

Multimedia Appendix 4 Top 10 most important acoustic feature dimensions in random forest models for suicide risk prediction by using high-level statistical function features.

## Abbreviations

FN: false negative
FNR: false negative rate
FP: false positive
HSF: high-level statistical function
MFCC: Mel Frequency Cepstral Coefficient
MFSC: mfcc_sma [13]_centroid
MP3: MPEG-1 audio layer 3
OpenSMILE: Open-Source Speech and Music Interpretation by Large-Space Extraction
PDP: partial dependence plot
PMSS: pcm_fftMag_spectralSkewness_sma_iqr1-3
RASTA: Reliable And Smooth Template Algorithm
SMA: simple moving average
SRRS: audSpec_Rfilt_sma [0]_stddevRisingSlope
TN: true negative
TP: true positive
WAV: waveform audio file

## Data Availability

Data cannot be publicly provided because of privacy concerns. Oral informed consent obtained from the study participants does not permit public sharing of the data. The principal investigator of this project (ZS) and the corresponding author (LY) have full access to the data and are responsible for its integrity. Further inquiries, including the study protocol, should be directed to these authors.
