# Supplementary material for: Acoustic Features for Identifying Suicide Risk in Crisis Hotline Callers: Machine Learning Approach
Source: J Med Internet Res. 2025 Apr 14;27:e67772. doi: 10.2196/67772 (PMC12038290; doi:10.2196/67772)
Supplement: Multimedia Appendix 1 [file jmir_v27i1e67772_app1.docx]

**Multimedia Appendix 1.** Basic acoustic features extracted with *librosa* included in the study.

| Feature name |
| --- |
| Duration |
| f0 |
| Short-time Energy |
| Zero Crossing Rate |
| MFCC1 |
| MFCC3 |
| MFCC4 |
| MFCC5 |
| MFCC6 |
| MFCC7 |
| MFCC8 |
| MFCC10 |
| MFCC11 |
| MFCC12 |
| MFCC13 |
| MFCC14 |
| MFCC15 |
| MFCC16 |
| MFCC17 |
| MFCC18 |
| MFCC19 |
| MFCC20 |
| MFCC21 |
| MFCC22 |
| MFCC23 |
| MFCC24 |
| MFCC25 |
| MFCC26 |
| MFCC27 |
| MFCC28 |
| MFCC29 |
| MFCC30 |
| MFCC31 |
| MFCC32 |
| MFCC33 |
| MFCC34 |
| MFCC35 |
| MFCC36 |
| MFCC37 |
| MFCC38 |
| MFCC39 |
| Formant 1 |
| Formant 2 |
| Formant 3 |
| Bandwidth of Formant 1 |
| Bandwidth of Formant 2 |
| Bandwidth of Formant 3 |
| Jitter |
| Shimmer |

^a^f0: Fundamental Frequency

^b^MFCC: Mel-frequency cepstral coefficient
