# Supplementary figures and images for: Acoustic Features for Identifying Suicide Risk in Crisis Hotline Callers: Machine Learning Approach
Source: J Med Internet Res. 2025 Apr 14;27:e67772. doi: 10.2196/67772 (PMC12038290; doi:10.2196/67772)

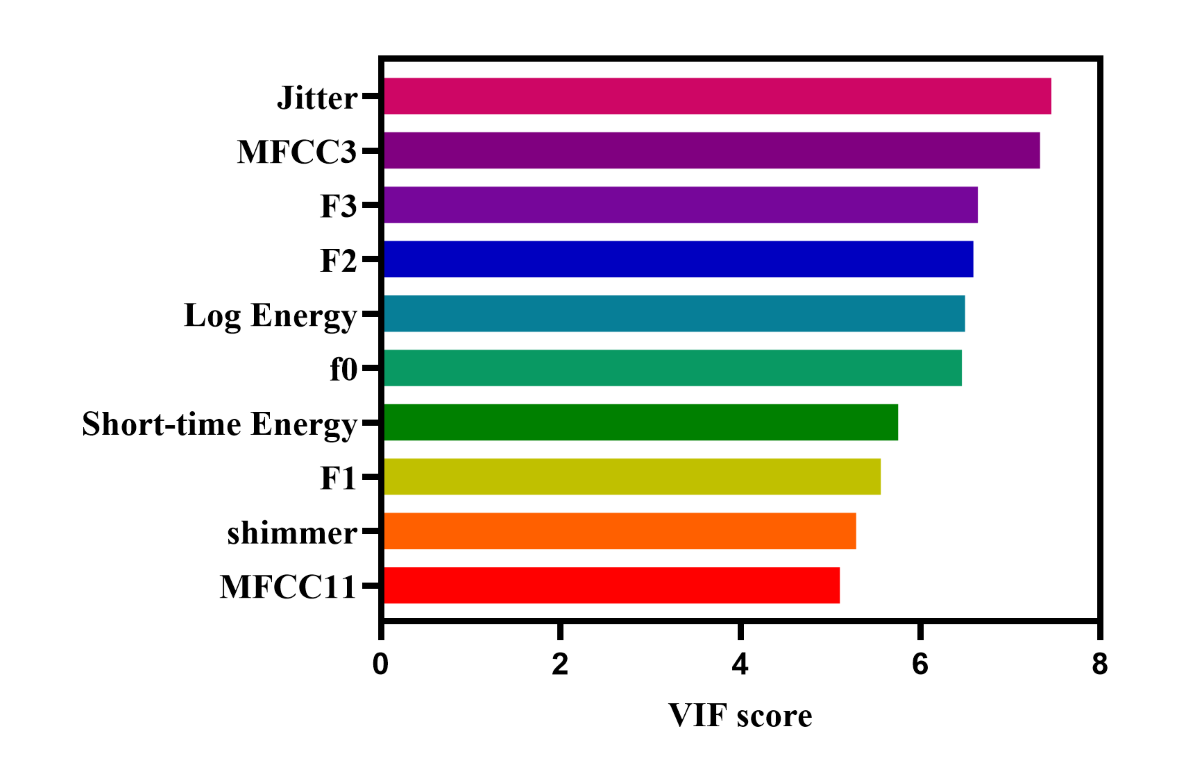

Supplement: Multimedia Appendix 2 [file jmir_v27i1e67772_app2.png]

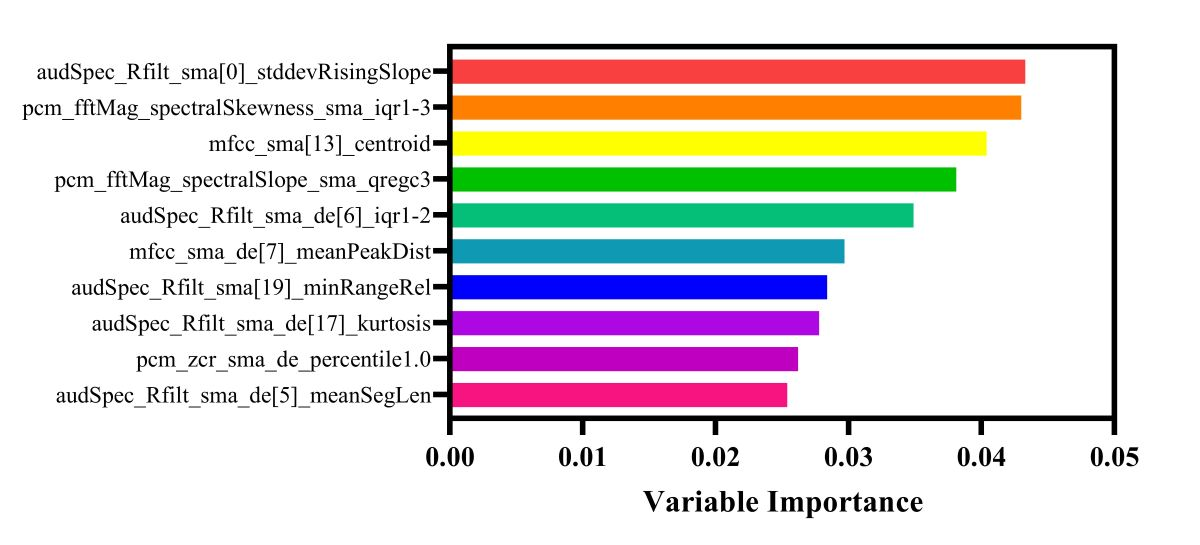

Supplement: Multimedia Appendix 4 [file jmir_v27i1e67772_app4.png]
