# Supplementary material for: Acoustic Features for Identifying Suicide Risk in Crisis Hotline Callers: Machine Learning Approach
Source: J Med Internet Res. 2025 Apr 14;27:e67772. doi: 10.2196/67772 (PMC12038290; doi:10.2196/67772)
Supplement: Multimedia Appendix 3 [file jmir_v27i1e67772_app3.docx]

**Multimedia Appendix 3.** Fifty dimensional features with the highest mutual information values in ComParE 2016.

| Feature name |
| --- |
| shimmerLocal_sma_minPos |
| pcm_fftMag_fband250-650_sma_de_posamean |
| pcm_RMSenergy_sma_de_minSegLen |
| audSpec_Rfilt_sma_de[12]_meanFallingSlope |
| audSpec_Rfilt_sma[0]_stddevRisingSlope |
| pcm_fftMag_psySharpness_sma_de_posamean |
| pcm_RMSenergy_sma_de_upleveltime50 |
| audSpec_Rfilt_sma[0]_range |
| pcm_fftMag_spectralSkewness_sma_minSegLen |
| mfcc_sma_de[10]_meanFallingSlope |
| pcm_fftMag_spectralCentroid_sma_quartile2 |
| audSpec_Rfilt_sma[22]_peakMeanAbs |
| pcm_fftMag_spectralRollOff75.0_sma_de_peakRangeAbs |
| mfcc_sma[6]_risetime |
| mfcc_sma[2]_pctlrange0-1 |
| pcm_fftMag_spectralSlope_sma_qregc3 |
| audSpec_Rfilt_sma_de[6]_iqr1-2 |
| audSpec_Rfilt_sma[12]_lpc4 |
| F0final_sma_de_qregc1 |
| mfcc_sma[13]_centroid |
| mfcc_sma[7]_minSegLen |
| mfcc_sma[10]_lpgain |
| mfcc_sma_de[2]_minSegLen |
| pcm_fftMag_spectralRollOff90.0_sma_quartile1 |
| audSpec_Rfilt_sma_de[16]_meanRisingSlope |
| pcm_fftMag_spectralRollOff90.0_sma_qregc1 |
| pcm_fftMag_spectralRollOff90.0_sma_meanRisingSlope |
| mfcc_sma_de[14]_percentile99.0 |
| audSpec_Rfilt_sma[21]_iqr1-2 |
| pcm_zcr_sma_de_percentile1.0 |
| audSpec_Rfilt_sma[1]_stddevFallingSlope |
| audSpec_Rfilt_sma[8]_flatness |
| pcm_fftMag_fband1000-4000_sma_de_iqr1-2 |
| audSpec_Rfilt_sma[19]_minRangeRel |
| mfcc_sma[8]_upleveltime75 |
| mfcc_sma[2]_qregc1 |
| audSpec_Rfilt_sma[19]_upleveltime50 |
| pcm_fftMag_spectralCentroid_sma_lpc1 |
| audSpec_Rfilt_sma_de[5]_meanSegLen |
| audSpec_Rfilt_sma_de[17]_kurtosis |
| shimmerLocal_sma_flatness |
| audSpec_Rfilt_sma_de[14]_stddev |
| jitterDDP_sma_de_amean |
| mfcc_sma_de[2]_stddevFallingSlope |
| pcm_fftMag_spectralSkewness_sma_iqr1-3 |
| audSpec_Rfilt_sma_de[0]_upleveltime75 |
| mfcc_sma[5]_quartile1 |
| mfcc_sma_de[7]_meanPeakDist |
| mfcc_sma[5]_peakMeanAbs |

^a^SMA: Short-term Moving Average

^b^minPos: Minimum Position

^c^PCM: Pulse Code Modulation

^d^fftMag: Fast Fourier Transform Magnitude

^e^fband: Frequency Band

^f^de: Delta Energy

^g^posamean: Positive Area Mean

^h^RMS: Root Mean Square

^i^audSpec: Auditory Spectrum

^j^Rfilt: Reduced Filter

^k^stddev: Standard Deviation

^l^upleveltime: Time to Up Level

^m^MFCC: Mel-frequency Cepstral Coefficients

^n^pctlrange: Percentile Range

^o^IQR: Interquartile Range

^p^LPC: Linear Predictive Coding

^q^F0final: Final Fundamental Frequency

^r^lpgain: Linear Prediction Gain

^s^ZCR: Zero Crossing Rate

^t^minRangeRel: Minimum Range Relative

^u^qregc: Quantile Regression Coefficients

^v^jitterDDP: Jitter (Delta Delta Pitch)
